# Digital adoption and efficiency in the maritime industry

**DOI:** 10.1186/s41072-022-00111-y

**Published:** 2022-05-09

**Authors:** Dimitris Gavalas, Theodoros Syriopoulos, Efthimios Roumpis

**Affiliations:** 1 Department of Shipping, Trade and Transport, School of Business, University of the Aegean, Korai 2A, 82312 Chios, Greece; 2 Department of Ports Management and Shipping, School of Economics and Political Sciences, National and Kapodistrian University of Athens, Psachna Evias, Greece; 3 Audencia Business School, Nantes, France

**Keywords:** Maritime industry, Shipping firms, Digitalization, ICT, Efficiency

## Abstract

The COVID-19 pandemic has augmented pre-existing digitalization and environmental trends. In the maritime industry, one of the marked impacts of the pandemic is how the regard for technology has changed. There is now greater appetite and acceptance of digital solutions across the industry. This study investigates the ways the adoption of a series of digital technologies impact shipping firms’ efficiency, that will shed light on how industry stakeholders may derive value from data solutions, for making better operational decisions. We use cross-country firm-level data to evaluate the efficiency effects of maritime industry-level digital adoption. The results provide robust proof that working in a digitalized ecosystem is a way to promote efficiency, though not to the same extent across shipping firms and divisions. Impacts are relatively stronger in water transport activities than warehousing/support activities for transportation. Digital technologies may add to the growing diffusion in efficiency across shipping firms.

## Introduction

The maritime industry like any other service industry copes with the difficulties of worldwide competition and increasing needs for efficiency. Together with the worries for human safety and environmentally safe operations, the strategic aspects of its service excellence consist of operations and management efficiency which are pinpointed by the results of service efficiency and enabled by technology applications for process efficiency. In the maritime industry, one of the marked impacts of the COVID-19 pandemic is how the regard for technology has changed. The pandemic has augmented pre-existing digitalization and environmental trends. There is now greater appetite and acceptance of digital solutions across the industry. A data driven transformation that extends beyond ship-owners, ship-managers to charterers, financiers, and insurers is very much underway.

There is extensive consent that digital technologies can generate positive impacts on efficiency at the firm and industry level. However, efficiency benefits from digital adoption are not guaranteed. They are subject to firms’ organizational capital and management skills, including their capacity to implement matching investments and modernizations to enhance business practices and systematize a number of routine tasks. Moreover, efficiency benefits can take time to materialize (Sorbe et al. 2019). Apart from the enduring attempts to accomplish more sustainability in worldwide transportation, there is also the digital revolution as an additional obstacle. Ever since the outbreak of the COVID-19 pandemic, the focus of management attention might have changed towards short-term adjustments to cope with the completely unpredicted and unforeseen new situation. This study evaluates maritime digital adoption and efficiency maturity, that will shed light on how industry stakeholders may derive value from data solutions, for making better operational and strategic^1^ decisions.

The authors have used cross-country firm-level data to evaluate the efficiency effects of maritime industry-level digital adoption. They resulted in robust proof that working in a digitalized ecosystem is a way to promote efficiency, though not to the same extent across firms and industries. The problem of technological change is also not the same should a shipping firm be in an early or late adopting stage of current technologies; the same situation occurs for large and financially powerful versus small and growing firms. Their awareness of value and risk are somewhat different, which afterwards influences their technology replacement decisions. This study pursues—among others—whether the heterogeneity of adoption levels and adoption effects across shipping firms and divisions may be a factor to justify why cumulative advances from digital adoption appear to be inadequate to counteract other aspects affecting the efficiency deceleration.

Additionally, this study pursues to answer the question of whether efficiency gains are greater for high efficiency maritime firms, suggesting that digital adoption in the maritime industry impacts the growing efficiency diffusion across firms in this industry; contrariwise, do efficiency benefits hinge on firm size? Other questions which are dealt within the scope of this study include: (1) whether the efficiency advantages of adoption are considerably frustrated by skill and occupational deficiencies, and (2) whether digital adoption in the two available under study Eurostat divisions (NACE Rev.2)^2^ are generally considered more valuable in water transport than warehousing and support activities for transportation firms.

The structure of this study is as follows: Section “Information technologies (IT) intensity and operational efficiency” discusses the most relevant previous studies. We proceed with section “Methodological approach”, presenting the methodological approach, using an error correction model. Subsequently, in section "Interpreting the results" of measuring the baseline model are demonstrated, running an ordinary least squares (OLS) regression. Finally, concludes section "Conclusion and discussion".

## Information technologies (IT) intensity and operational efficiency

### Literature review

Bartel et al. (2007) investigated the consequences of modern IT on efficiency by building a data set on plants in one industry and examining numerous plant-level processes over which IT could stimulate efficiency progress. They resulted in that (1) plants implementing innovative equipment modify their operational policies by delivering more tailored products, (2) IT investments advance the competence of the manufacturing cycle, and (3) adoption of capital equipment driven by IT leads to growth on skill requirements of machine operators, and with the implementation of novel human resource policies to uphold these skills. In their study, Brynjolfsson et al. (2008) classified a number of industry-level shifts that took place in the mid-1990s, claiming to be consistent with an increased use of IT. They argued a considerable rise in instability, as quantified—among others—by the average intra-industry rank change in sales. A series of firm- and industry-level studies argue about positive links concerning investment in digital technologies and efficiency. Moreover, Munch et al. (2018) compiled a report which assesses the literature on technological transformation and its consequences for individual workers, firm productivity, and the nature of work.

The extensive perception of technology denotes the state of awareness on how to transform resources into outputs. This comprises the everyday use and application to business processes or products of technical procedures, systems, devices, skills and practices. Nevertheless, the term ‘technology’ is meant to signify precisely the technical capability to put in motion and control several material transformation procedures. Competences in this area draw on the awareness and know-how of a shipping firm’s staff, accrued understanding in using technologies and the capacity to use assets (ships in this case) entrenching technology (Galindo-Rueda et al. 2020).

Several other studies have concluded into different results regarding the relationship between IT and operational efficiency. This generally confusing situation reveals that connections among digitalization and efficiency seem complicated, while their empirical detection being demanding as well. After carefully studying the literature and analyzing our study’s results, the main driver seems to be that digital technologies typically uphold efficiency, however when combined with other components. In this context, Bartelsman et al. (2017) discovered no significant impact of broadband access on within-firm efficiency. Nevertheless, they resulted in a positive effect at the aggregate level, suggesting positive impact after employing the reallocation indicator.^3^ De Stefano et al. (2014) employed a fuzzy regression discontinuity model to examine the consequences of ADSL broadband internet on the firm efficiency. The discontinuity occurred for a 5-year time period, based on discrepancies in the timing of broadband accessibility amongst two telecom suppliers in a specific region of the UK. Using this empirical model, they argued that (1) better access to broadband infrastructure substantially strengthens the prospect of adoption, and (2) no strong evidence had been found that utilizing broadband had an impact on firm efficiency. A similar reluctance to results can be found in Acemoglu et al. (2014), where after employing US firm-level data over 1977–2007, they argued that there was no effect of IT intensity on manufacturing efficiency, with the exception of the computer-producing industry.

During the last fifteen years, there have been a series of studies focusing or emphasizing on the importance of the information/digital technologies in the maritime industry, through applying several methodologies and approaches (i.e. Gavalas et al. 2021; Gavalas and Syriopoulos 2015; Poulis et al 2013; Siror et al. 2011; Nikitakos and Lambrou 2007). Recently, Papathanasiou et al. (2020) empirically examined the real and potential initiation of blockchain technology (BT) adoption in the Greek shipping industry and defined the motives regarding the adoption scale. They found that (among others) the operations and logistics tasks could be vastly enhanced through BT, whereas the incorporation of BT with enterprise resource planning systems could be capable of totally converting the way daily operations are carried out. Yang (2019) specified the notion of maritime shipping digitization like applying disruptive technologies to minimize transportation expenditures and structure global trade expectations, offering firms a greater level of competitive advantage. The study employed the technology acceptance model, which investigates how users become eager to acknowledge the use of new technologies, by evaluating the intention to practice innovative technologies from two perspectives: the user-friendliness of the innovative technology and its level of practical application.

Moreover, Tan and Sundarakani (2020) employed a context for a freight consolidation firm to adopt BT, after exploring the difficulties faced by a global shipping company and discovering the use of BT to boost the competitiveness and sustainability of freight booking procedures. Their study is principally based on Technology Acceptance Model (TAM) theory. Additionally, Pu and Lam (2020) recommended that the decisive objective of BT applications is to attain lean process by means of dropping paperwork, improving data allotment, and automating procedures. They employed a conceptual framework to deliver a holistic view of BT adoption in the maritime industry by responding to questions of why BT can be pertained, via which means it could be pertained, and define the market participants.

Another study of Balci and Surucu-Balci (2021) revealed the underlying interaction between BT adoption barriers in Computer Information Technology (CIT) and uncovered the most prominent stakeholders for the adoption. A total of eight barriers were identified, and their relationships were uncovered through Interpretive Structural Modelling (ISM) approach, after a total of 30 experts completed the study’s survey. The barriers identified were: lack of government regulations, lack of trust towards BT, privacy/business information sharing concerns in blockchain platforms, lack of knowledge/understanding about BT, lack of support from influencing stakeholders, resistance of some stakeholders to adopt, perceived resource and initial capital requirements, and lack of early adopters.

Although these studies concentrate on digitalization approaches under several application features, to the best of our knowledge there is no study using cross-country firm-level data to evaluate the efficiency impact of industry-level digital adoption in the maritime industry. Such combination is a means of alleviating endogeneity matters, whereas letting to cover both within-firm and spillover effects of adoption. The common emphasis on certain digital technologies gives space to a more sophisticated classification.

### Setting the scene

Ever since the 2008 global financial crisis, the world economy has been confronting bumpy growth expectations. Specifically, trade growth endures restrained, while long-term growth fundamentals persist to be weak. Such economic conditions that may cause a decline in profits, are progressively converting into long-term underlying moves in the global maritime industry. Moreover, the COVID-19 pandemic disturbed maritime transport, though the outcome has been considered by the practitioners less damaging than at first feared. The shock in the H1/2020 triggered maritime trade to reduce by 3.8%. But in 9M/2020 a recovery occurred for both containerized trade and dry bulk commodities; the same did not happen for tanker sector (IMF 2021; Gavalas et al. 2022).

Moreover, technological improvements have allowed shipping and ports to maintain operations while reducing interface and physical contact. Shipping firms are leveraging improvements in both hardware and software to optimize their operations. With technology distorting traditional industry limitations, new players are emerging to deliver technology-driven solutions, providing more added value compared to traditional business models. New technologies have also encouraged the rise of e-commerce which has converted consuming behaviors. The increase in e-trade has boosted the demand for logistics facilities and warehousing that are digitally supported (Waypoint Digital 2017; Kramberger 2016). One of the contributions of this study is to evaluate the current efficiency impact of industry-level digital adoption in the maritime industry.

Regarding labor trends, all countries and industries confront different ambiguities over the next decades, stemming from shifts in crucial sectors like shipping, and by advances in technology that will alter the way vacancies are performed and assets are managed (UNCTAD 2021). It is therefore necessary for this study to answer on the skills which will be needed for the future and the way to contribute to digital opportunities, while noticing not to dispose components that have influenced to previous success and shall persist being vital in future.

Another contribution of this study is estimating the degree of digitalization varying between subsectors (divisions in this case) and firms in the maritime industry by examining the heterogeneous impact of digital adoption on efficiency quartiles and various firm size categories. An interesting report by Waypoint Digital (2017) revealed that the ship management sector had showed how digital tools such as data analytics, smart sensors, and the Internet-of-Things can be attached to improve operational efficiency in fields such as “predictive vessel maintenance, bunker fuel optimization, and global fleet monitoring”. However, an appealing result of that report was that even though the prospects offered by digitalization, relevant investments by shipping firms had remained low at that time.

## Methodological approach

### Setting the model

Leading organizations use a variety of measures to evaluate their performance. A common indicator of organization performance is productivity. In basic terms, productivity relates to the output of goods and services divided by its input. The measurement of productivity can be employed by two approaches: the multi factor productivity (MFP) and single factor productivity. MFP is the ratio of total outputs to total inputs, whereas single factor productivity measures the ratio of outputs to a single category of inputs (Stone et al. 2020). How may one consider combining the technological dissemination and innovation? In an attempt to answer this, the OECD multi-factor productivity (MFP) has been applied in this study. According to OECD (2021), MFP reflects the overall efficiency with which labor and capital inputs are used together in the production process. Changes in MFP reflect the effects of changes in management practices, brand names, organizational change, general knowledge, network effects, spillovers from production factors, adjustment costs, economies of scale, the effects of imperfect competition and measurement errors. Growth in MFP is measured as a residual, i.e. that part of GDP growth that cannot be explained by changes in labor and capital inputs. In simple terms therefore, if labor and capital inputs remained unchanged between two periods, any changes in output would reflect changes in MFP.^4^ Econometrically, we have followed the studies of Bourlès et al. (2013), and Gal et al. (2019), where (among other studies) MFP is considered to follow an error correction model of the form:

1 $$\Delta MFP_{f, d, c, t } = a_{1} \,\Delta MFP_{Frontier d, t } + a_{2} \,Distance_{f, d, c, t - 1 } + \beta \,DA_{{ d, c, \overline{t}}} + \gamma \,Z_{f, d, c, t } + \delta_{c,t} + \delta_{i} + \varepsilon_{c,t} ,$$

where ${\Delta MFP}_{f, d, c, t }$ denotes the shift in the logarithm of MFP of shipping firm *f*, which operates in division *d* and country *c*, in year *t*. MFP growth of firm *f* is presumed to hinge on MFP growth of the efficiency frontier (${\Delta MFP}_{Frontier d, t}$), which is described as the average MFP among the 5% most productive firms in division *d* and year *t* across the countries in the sample, and on the lagged distance to the frontier (${Distance}_{f, d, c, t-1}$ = ${MFP}_{Frontier d, t-1}$ − ${MFP}_{f,d, t-1}$).

Frontier shipping firms have been excluded from the sample to avoid endogeneity concerns. According to previous studies (i.e. Berlingieri et al. 2020; McGowan et al. 2017), one should presume α_1_ to be positive but below 1, suggesting that innovation at the frontier advantages shipping firms but only partially, whereas α_2_ to be positive, suggesting that shipping firms below the frontier value from the theory of convergence, meaning that they might possibly replicate the other firms’ methods. Nevertheless, the degree the frontier shifts might mean efficiency convergence or divergence among shipping firms.

Coefficient β, which depicts the effect of industry-level digital adoption on firm-level efficiency growth is of high importance. ${DA}_{ s, c, \overline{t} }$ embodies the portion of firms in division *d* and country *c,* which use a specific digital technology averaged over the period 2015–2020. The impact of distinct digital technologies has been measured in distinct identical regressions. Moreover, following Andrews et al. (2018), technologies combined effect has been measured employing a composite indicator of adoption, being built as the principal component of the five variables, signifying the implementation of different digital technologies.

A question to answer here would be about the profile of shipping firms and divisions which mainly benefit after the adoption of digitalization and what might the prospective interconnection with other factors be. To answer this, the digital adoption variable has been interrelated (1) with a categorical variable breaking water transport, and warehousing and support activities for transportation, and (2) with a variable encapsulating the average routine intensity of duties in each shipping industry, following Zhang and Tang (2021). Furthermore, in an effort to evaluate which shipping firms benefit more from the dissemination of digital technologies, the digital adoption variable has been interrelated with two categorical variables dividing the sample into size and efficiency classes, following Gal et al. (2019).

As digital adoption is detected only for one or two years in the period of interest, the regression depends on the average of the digital adoption variable over the available years (${DA}_{ d, c, \overline{t} }$), meaning that adoption does not fluctuate over time. From the one hand, this might put identification in danger, from the other hand prospective endogeneity issues could be alleviated, without omitting the observation of lagged benefits of digital adoption. As far as the vector of control variables ${Z}_{f, d, c, t}$ is concerned, it captures firm size, age, division, and country-year fixed effects. Nevertheless, such an empirical framework has the advantage of considering plausible firm heterogeneities and crucial drivers of efficiency (firm-specific); this leads us to a more robust framework than an industry-level one (Wang 2018).

However, there are a few drawbacks to be unfolded. Initially, endogeneity (even being small or negligible) might endure. This is plausible to happen when factors being unobserved influence simultaneously adoption levels in a division and efficiency growth rates of the firms in each division; this means that the structured model has failed to explain this by division and country-year fixed effects and by the supplementary control variables. Second, the top 5% efficiency frontier being used for shipping firms in each division might cause understatement of the impact of innovative technologies, that some firms might be pioneers in adopting them. Third, based on the convergence theory previously discussed, some shipping firms might manage to fill in the gap by adopting digital technologies that more advanced firms^5^ have previously adopted; this could be explained via the efficiency gap variable instead of the digital adoption (Zhang and Tang 2021).

### Blended firm and industry-level information

This study has merged a series of industry-level sources as far as routine intensity, digital adoption and occupational shortages are concerned, with firm-level data about efficiency growth. Information about digital adoption has been taken from the Eurostat “ICT usage in enterprises (isoc_e)” including country and industry components. Data given in this domain are collected on a yearly basis by the National Statistical Institutes or Ministries and are based on the annual Eurostat model questionnaires on ICT usage and e-commerce in enterprises. Furthermore, it supports measuring the implementation of one of the six priorities for the period 2019–2024 of European Commission—A Europe fit for the digital age. The coverage includes: ICT systems and their usage in enterprises, use of the Internet and other electronic networks by enterprises, e-commerce, e-business processes and organizational aspects, ICT competence in the enterprise and the need for ICT skills, barriers to the use of ICT, the Internet and other electronic networks, e-commerce and e-business processes, ICT security and trust, access to and use of the Internet and other network technologies for connecting objects and devices (Internet of Things), access to and use of technologies providing the ability to connect to the Internet or other networks from anywhere at any time (ubiquitous connectivity), use of Big data analysis, use of 3D printing, use of robotics, and use of Artificial Intelligence (Eurostat 2021).

The question arising here is about the subset of indicators to use. After carefully reading the literature, as well as the necessary documentation stemming from the European Commission (i.e. European Commission 2020), this study ended up following indicators similar to Andrews et al. (2018), and Gal et al. (2019), namely (1) Sophisticated Cloud Computing, (2) Broadband Internet Connection, (3) Enterprise Resource Planning, (4) Customer Relationship Management, and (5) Ecommerce Website.^6^ Table 1 denotes the description of the above variables (digital solutions). The aforementioned variables come in annual data series, stemming from Eurostat comprehensive database (Digital economy and society statistics—households and individuals) and their coverage is from 2015 to 2020.

**Table 1:** Description of variables

Digital technologies	Eurostat description	ExpVariable
Sophisticated Cloud Computing	Buy high CC services (accounting software applications, CRM software, computing power)	E_CC_HI
Broadband Internet Connection	The maximum contracted download speed of the fastest fixed line internet connection is at least 500 Mb/s but less than 1 Gb/s	E_ISPDF_500_1G
Enterprise Resource Planning	Enterprises who have ERP software package to share information between different functional areas	E_ERP1
Customer Relationship Management	Enterprises using software solutions like Customer Relationship Management (CRM)	E_CRM
Ecommerce Website	Website has possibility for visitors to customise or design online goods or services	e_webctm

Efficiency and other firm-level variables come from Bureau van Dijk, Orbis database (a Moody’s Analytics company), based on the information structure steps depicted in Gal (2013), Gopinath et al. (2017), and Andrews et al. (2018). A crucial issue to deal with was the data cleansing procedure to convert the financial data to a database appropriate for economic analysis. To ensure such, this study initially proceeded with comparability indications of nominal variables across countries and over timeframe, then received additional indicators, and of course kept solely shipping company accounts with valid and applicable information for this paper objective. The study acquired efficiency as a residual from measuring value-added based production tasks, for each detailed division independently, applying the control function methodology relying on transitional inputs to alleviate the endogeneity of input selections (Wang 2018). The sample has been limited to shipping firms having an average of at least 10 employees to meet the reference group of the division-level digital adoption indicator.

As far as the control variables at the industry level are concerned, the study of Pak and Schwellnus (2019) has been followed, who provide information of the routine content of occupations, based on the degree of independence and freedom in scheduling and establishing the duties to be accomplished on the occupation as a proxy for non-routine content. Moreover, the indicators measuring skill shortage and surplus rely on the OECD Skills for Jobs database (OECD 2018a) and are constructed on the basis of signals extracted from five sub-indices: wage growth, employment growth, hours worked growth, unemployment rate, and under-qualification growth. The indicators encompass seven sets of skills, of which we use the following ones: (1) complex problem-solving skills, including complex problem solving, (2) technical skills, including operations analysis, technology design, equipment selection, installation programming, operation monitoring, operation and control, equipment maintenance, troubleshooting repairing, quality control analysis, (3) systems skills, including judgment and decision making, systems analysis, systems evaluation, and (4) resource management skills, including time management, management of financial resources, management of material resource, management of personnel resources. As the OECD Skills for Jobs database includes seven big sets of skills, we focused on skills that are expected to be most corresponding to a shipping firm digital adoption. Our joint dataset extends over the EU 27 countries and 2 available divisions over 2015–2020 (Table 2).

**Table 2:** Descriptive statistics

	Mean	Median	Bottom decile	Top decile	Standard deviation
*Digital variables*					
Broadband Internet Connection	0.3412	0.3111	0.1451	0.6310	0.1743
Enterprise Resource Planning	0.3321	0.3016	0.1022	0.5986	0.1712
Customer Relationship Management	0.3316	0.2788	0.1369	0.5566	0.1707
Ecommerce Website	0.2359	0.1869	0.0643	0.4964	0.1769
Sophisticated Cloud Computing	0.1245	0.1144	0.0316	0.2777	0.1216
1st principal component	0.8466	0.3469	− 1.6145	4.2648	2.2730
*Firm-level variables*					
MFP growth	0.0097	0.0095	− 0.2411	0.2665	0.2551
Frontier growth	0.0185	0.0174	− 0.213	0.0648	0.0267
Gap to frontier	17,690	1.5669	0.7553	2.1247	0.6119
Age	19.4581	17	3	39	15.6541
Employees	3.4684	3.2451	2.6840	4.6496	1.0556
Capex	10.5596	10.5166	8.1236	11.1025	1.6499
*Industry-level variables*					
Routine intensity	− 0.1016	0.0215	− 0.6953	0.3466	0.3517
Skill shortages	− 0.0448	− 0.0236	− 0.2148	0.2080	0.2153
Resource management skills	0.0038	0.0055	− 0.0270	0.0285	0.0188
Management of personnel resources	0.0045	0.0057	− 0.0245	0.0385	0.0349
Computer and electronics	0.0156	0.0113	− 0.0322	0.0745	0.0483
Technical skills	− 0.0014	− 0.0007	− 0.0176	0.0146	0.0272

MFP, employees, and Capex are measured in logarithms

The digital technologies description has been displayed in Table 1. Additionally, in regard to the shipping firm-level variables, ‘frontier growth’ is denoted as the average growth of the top 5% shipping firms in each division-year cell, ‘gap to frontier’ means the shipping firms’ lagged distance to the frontier, ‘age’ is the shipping firms’ age, ‘employees’ defines the shipping firms’ number of employees (log), and capex presents the capital expenditures (log).

The efficiency gains from digital adoption seem greater in industries that are intensive in routine tasks. This validates that streamlining or automating routine assignments is one of the channels through which digital adoption enhances efficiency. Though this could put up questions for policy in terms of job losses, digital adoption is also predisposed to creating new jobs due to their complementarity with skilled labor (OECD, 2019), this being an issue for further research.

## Interpreting the results

In the table below (Table 3), we demonstrate the results of measuring the baseline MFP model, running an OLS regression. All coefficients show the expected sign and significance; namely all digital technologies observed are positively and significantly correlated to MFP growth. In column 1 for example, we see that approximately 20% of expansions in “frontier growth” are forwarded to the average shipping firm and 10% of the “gap to frontier” is covered through convergence. Moreover, the last column displays findings for the 1st principal component of the five technologies. All regressions include division and country-year fixed effects and are grouped at the country-division level. Shipping firms being at the division-year threshold have not been included in the regressions. Regressions are based on firm-level data from EU 27 countries and 2 divisions, over the period 2015–2020, for shipping firms with more than 10 employees. To expand exposure, unweighted averages of each digital technology indicator have been used over the period 2015–2020. Furthermore, the degree that the regression model fits the observed data (R-squared) comes in line with Haider et al. (2021), Ciarli et al. (2021), Gal et al. (2019), Andrews et al. (2016).

**Table 3:** OLS Baseline Results

	Baseline	Broadband Internet Connection	Enterprise Resource Planning	Customer Relationship Management	Ecommerce Website	Sophisticated Cloud Computing	1st Principal Component
Frontier growth	0.211***	0.220***	0.215***	0.217***	0.208***	0.228***	0.231***
	(0.0312)	(0.0364)	(0.0367)	(0.0368)	(0.0316)	(0.0379)	(0.0386)
Gap to frontier	0.101***	0.101***	0.102***	0.104***	0.108***	0.109***	0.109***
	(0.0101)	(0.0107)	(0.0107)	(0.0126)	(0.0119)	(0.0123)	(0.0128)
Age	0.0001***	− 0.0002***	0.0003***	− 0.0002***	− 0.0002***	0.0002***	− 0.0003***
	(5.17e-05)	(5.27e-05)	(5.43e-05)	(5.81e-05)	(5.51e-05)	(5.64e-05)	(6.03e-05)
Employees	0.0221***	0.0213***	0.0216***	0.0218***	0.0218***	0.0219***	0.0227***
	(0.0024)	(0.0026)	(0.0025)	(0.0021)	(0.0024)	(0.0027)	(0.0028)
Digital Technology		0.138***	0.101**	0.173***	0.0853**	0.0385	0.0158***
		(0.0337)	(0.0359)	(0.0362)	(0.0428)	(0.0564)	(0.0033)
R-squared	0.057	0.054	0.057	0.057	0.054	0.058	0.058

Table demonstrates the measurements of the baseline equation where MFP growth (dependent variable) has been regressed on average MFP growth of the 5% shipping firms with highest MFP in each division-year, the firm’s gap to frontier, age, number of employees, along with adoption rates of individual digital technologies. ***, ** and * correspond to *p* < 0.01, *p* < 0.05 and *p* < 0.1 respectively

Something notable here is the various degrees of efficiency and digital adoption among the two available shipping divisions. To find this we have incorporated NACE Rev.2, Section H^7^—Transportation and Storage data, and precisely Divisions 50 & 52 (Eurostat 2008). Division 50 includes the transport of passengers or freight over water, whether scheduled or not. Also included are the operation of towing or pushing boats, excursion, cruise or sightseeing boats, ferries, water taxis etc. Although the location is an indicator for the separation between sea and inland water transport, the deciding factor is the type of vessel used. Transport on sea-going vessels is classified in groups 50.1 and 50.2, while transport using other vessels is classified in groups 50.3 and 50.4. This division excludes restaurant and bar activities on board ships, if carried out by separate units. Division 52 includes warehousing and support activities for transportation, such as operating of transport infrastructure (e.g. airports, harbors, tunnels, bridges, etc.), the activities of transport agencies and cargo handling (Table 4).

**Table 4:** Shipping divisions used in the model

Section H – Transportation and Storage					
NACE Rev. 2 Division	Group	Class	Description	Detailed description	ISIC Rev. 4 Division
50			Water transport		
	50.1		Sea and coastal passenger water transport	This group includes the transport of passengers on vessels designed for operating on sea or coastal waters. Also included is the transport of passengers on great lakes etc. when similar types of vessels are used	
		50.10	Sea and coastal passenger water transport	This class includes, transport of passengers over seas and coastal waters, whether scheduled or not (operation of excursion, cruise or sightseeing boats, operation of ferries, water taxis etc.), renting of pleasure boats with crew for sea and coastal water transport (e.g. for fishing cruises)	5011
	50.2		Sea and coastal freight water transport	This group includes the transport of freight on vessels designed for operating on sea or coastal waters. Also included is the transport of freight on great lakes etc. when similar types of vessels are used	
		50.20	Sea and coastal freight water transport	This class includes transport of freight over seas and coastal waters, whether scheduled or not (transport by towing or pushing of barges, oil rigs etc.), and renting of vessels with crew for sea and coastal freight water transport	5012
	50.3		Inland passenger water transport	This group includes the transport of passengers on inland waters, involving vessels that are not suitable for sea transport	
		50.30	Inland passenger water transport	This class includes transport of passengers via rivers, canals, lakes and other inland waterways, including inside harbours and ports, renting of pleasure boats with crew for inland water transport	5021
	50.4		Inland freight water transport	This group includes the transport of freight on inland waters, involving vessels that are not suitable for sea transport	
		50.40	Inland freight water transport	This class includes transport of freight via rivers, canals, lakes and other inland waterways, including inside harbours and ports, and renting of vessels with crew for inland freight water transport	5022
52			Warehousing and support activities for transportation	This division includes warehousing and support activities for transportation, such as operating of transport infrastructure (e.g. airports, harbours, tunnels, bridges, etc.), the activities of transport agencies and cargo handling	
	52.1		Warehousing and storage		
		52.10	Warehousing and storage	This class includes operation of storage and warehouse facilities for all kinds of goods (operation of grain silos, general merchandise warehouses, refrigerated warehouses, storage tanks etc.), storage of goods in foreign trade zones, and blast freezing	5210
	52.2		Support activities for transportation	This group includes activities supporting the transport of passengers or freight, such as operation of parts of the transport infrastructure or activities related to handling freight immediately before or after transport or between transport segments. The operation and maintenance of all transport facilities is included	
		52.22	Service activities incidental to water transportation	This class includes activities related to water transport of passengers, animals or freight (operation of terminal facilities such as harbours and piers, operation of waterway locks etc., navigation, pilotage and berthing activities, lighterage, salvage activities, and lighthouse activities)	5222
		52.24	Cargo handling	This class includes loading and unloading of goods or passengers’ luggage irrespective of the mode of transport used for transportation, stevedoring and loading and unloading of freight railway cars	5224
		52.29	Other transportation support activities	This class includes forwarding of freight, arranging or organising of transport operations by rail, road, sea or air, organisation of group and individual consignments (including pickup and delivery of goods and grouping of consignments), issue and procurement of transport documents and waybills, activities of customs agents, activities of sea-freight forwarders and air-cargo agents, brokerage for ship and aircraft space, goods-handling operations, e.g. temporary crating for the sole purpose of protecting the goods during transit, uncrating, sampling, weighing of goods	5229

Afterwards, we proceeded with regressing firm-level MFP growth of the top 5% frontier shipping firms in each division-year, the firm’s gap to this frontier, age, number of employees, and eventually the interface amongst digital adoption rates and a dummy for each division. Table 5 below describes the values of the baseline equation where firm-level MFP growth is regressed on average MFP growth of the 5% shipping firms with highest MFP in each division-year cell, the shipping firm’s lagged gap to this efficiency threshold, and age/size. Moreover, the last column displays findings for the 1st principal component of the five technologies. All regressions include division and country-year fixed effects and are grouped at the country-division level. Shipping firms being at the division-year threshold have not been included in the regressions. Regressions are based on firm-level data from EU 27 countries and 2 divisions (NACE Rev.2, Section H), over the period 2015–2020, for shipping firms with more than 10 employees. To expand exposure, unweighted averages of each digital technology indicator have been used over the same period.

**Table 5:** Observing the segregation between “Water transport” & “Warehousing and support activities for transportation” Divisions

	Broadband Internet Connection	Enterprise Resource Planning	Customer Relationship Management	Ecommerce Website	Sophisticated Cloud Computing	1st Principal Component
Frontier growth	0.175***	0.115***	0.167***	0.169***	0.139***	0.181***
	(0.0366)	(0.0385)	(0.0416)	(0.0416)	(0.0463)	(0.0426)
Gap to frontier	0.118***	0.115***	0.117***	0.118***	0.120***	0.121***
	(0.0045)	(0.0045)	(0.0058)	(0.0053)	(0.0053)	(0.0054)
Age	− 0.0002***	0.0003***	− 0.0002***	− 0.0002***	0.0002***	− 0.0003***
	(5.29e-05)	(5.43e-05)	(5.41e-05)	(5.13e-05)	(5.02e-05)	(5.47e-05)
Employees	0.0264***	0.0226***	0.0223***	0.0237***	0.0243***	0.0266***
	(0.0025)	(0.0025)	(0.0013)	(0.0014)	(0.0026)	(0.0028)
Digital Technology	0.124***	0.101**	0.244***	0.176**	0.331***	0.0246***
*(Water transport)*	(0.0447)	(0.0359)	(0.0438)	(0.0496)	(0.137)	(0.0048)
Digital Technology	0.138***	0.0436	0.150***	0.0474	0.0569	0.0133***
*(Warehousing and support activities for transportation)*	(0.0321)	(0.0359)	(0.0325)	(0.0428)	(0.0564)	(0.0034)
R-squared	0.064	0.067	0.067	0.064	0.068	0.068

***, ** and * correspond to *p* < 0.01, *p* < 0.05 and *p* < 0.1 respectively. The last column shows results for the 1st principal component of the five technological tools

As observed in Table 5, greater firm-level efficiency is involved in the digital adoption process of water transport rather than warehousing and support activities for transportation; the only technological tool working the other way round seems broadband Internet Connection. This comes in line with Sorbe et al. (2019) argument that the efficiency gains from digital adoption seem greater in industries that are intensive in routine tasks. This validates that streamlining or automating routine assignments is one of the channels through which digital adoption enhances efficiency.

A few years ago, Akerman et al. (2015), assessed the impact of broadband internet on labor market outcomes and efficiency amongst different types of workers. They argued that such technology enhances (or deteriorates) the labor market outcomes and efficiency of skilled (or unskilled) workers, and precisely reinforced skilled workers in executing nonroutine abstract assignments and substituted for unskilled workers in performing routine assignments. In 2016, Marcolin et al. (2016) used information to build an assessment model of the routine content of occupations, based on information of the magnitude to which employees can adjust the sequence in which they execute their tasks and decide the type of tasks to be completed according to the job description. Recently, Chevalier and Luciani (2018) after employing a data series built for 228 manufacturing industries in France between 1994 and 2007 argued that discriminating between low-tech and mid/high tech industries and between high-skilled and low-skilled employees stipulates an adequate level of heterogeneity to depict discrete conclusions for efficiency and employment where automation is more concentrated.

A question arising here is how to proxy routine intensity; to do so, we follow Marcolin et al. (2016) by employing the Routine Intensity Indicator (RII), developed on statistics from the OECD Programme for the International Assessment of Adult Competencies survey (OECD 2016). The RII index integrating the routine content of the task executed by an employee *i* employed in division *d* and position *p*, could be written as:

2 $$RII_{i,d,p } = w_{freq } Frequency_{i,d,p } + w_{elast } Elasticity_{i,d,p } + w_{proj } Projection_{i,d,p } + w_{man } Management_{i,d,p } ,$$

where $Frequency$, $Elasticity$, $Projection$, and $Management$ are the rates of recurrence with which employees may, respectively (1) decide the regularity of the assignments involved by the position, (2) adjust the subject of job description or how this is executed, (3) plan their own work duties, and (4) manage their own working time. When forming the RII, a weight $w$ which is independent on the employee, his/her division and position, may be correlated to each variable with the intention of providing additional or less amount of significance to the distinct routine features.

The OECD Programme for the International Assessment of Adult Competencies survey mentioned above, states that each of the defined variables is implicit by a scale of integer values fluctuating from 1 to 5 and expanded so that 1 signifies the least frequency of routine-intensive operation, and 5 the highest such frequency. The ensuing index RII is consequently rising in the frequency of routine intensive operations, namely the greater the value of the index, the more routine intensive the operation is deemed. As far as the weights are concerned, the four weights should sum to one, so that the support of the RII is the same as its composing variables, and the RII is tied amongst 1 and 5.

After running the regressions in Eq. 2, we result in digital adoption being closer linked to efficiency gains in divisions extremely intensive in routine tasks than elsewhere (Table 6). We tend to agree with Gal et al. (2019) upon the plausible interpretation of it, being the existence of wider scope for substitution between technology and labor in these divisions.

**Table 6:** Observing the segregation in line with division routine intensity

	Broadband Internet Connection	Enterprise Resource Planning	Customer Relationship Management	Ecommerce Website	Sophisticated Cloud Computing	1st Principal Component
Frontier growth	0.216***	0.215***	0.219***	0.214***	0.227***	0.233***
	(0.0383)	(0.0380)	(0.0378)	(0.0381)	(0.0372)	(0.0374)
Gap to frontier	0.101***	0.102***	0.104***	0.108***	0.108***	0.109***
	(0.0142)	(0.0140)	(0.0144)	(0.0143)	(0.0141)	(0.0146)
Age	− 0.0002***	0.0003***	− 0.0002***	− 0.0002***	0.0002***	− 0.0003***
	(6.18e-05)	(6.19e-05)	(6.23e-05)	(6.19e-05)	(7.11e-05)	(7.37e-05)
Employees	0.0184***	0.0180***	0.0223***	0.0180***	0.0229***	0.0234***
	(0.0035)	(0.0035)	(0.0023)	(0.0024)	(0.0036)	(0.0038)
Digital Technology	0.148***	0.0531	0.157***	0.103**	0.165*	0.0209***
	(0.0564)	(0.0531)	(0.0429)	(0.0571)	(0.115)	(0.0052)
Digital Technology * routine intensity	0.0157	0.116**	0.128*	0.174*	0.271**	0.0246***
	(0.0614)	(0.0601)	(0.0781)	(0.0609)	(0.117)	(0.0052)
R-squared	0.064	0.062	0.064	0.064	0.067	0.067

***, ** and * correspond to *p* < 0.01, *p* < 0.05 and *p* < 0.1 respectively. The last column shows results for the 1st principal component of the five technological tools

One more notable question to answer is whether skill shortages in a division might hinder digital adoption from bearing its maximum efficiency gains. Skill shortages occur when employers are incapable of recruiting workforce with the required set skills in the available labor market and at that time salary level and working conditions. Skill surpluses evolve in the opposing instance, when the supply surpasses the demand for a particular skill (OECD, 2018a). We examine this assumption by broadening the baseline model beyond to incorporate the interface among digital adoption and skill shortages. One possible drawback here could be that shipping firms in which the level of digital adoption is high might trigger skill shortages, provoking a serious endogeneity issue (Cedefop 2015). Nevertheless, according to Table 7, there seems to be no high correlation of technological tools and skill shortages in the available data set.

**Table 7:** Relationship between skill shortages and digital adoption

	System skills	Technical skills	Complex problem-solving skills	Resource Management
Broadband Internet Connection	0.0545***	0.0684***	0.0771***	0.0866***
Enterprise Resource Planning	− 0.0786***	0.0926**	− 0.0586***	− 0.0556***
Customer Relationship Management	− 0.0436***	0.0421***	− 0.0465***	− 0.0354***
Ecommerce Website	0.0576***	0.0486***	0.0417***	0.0384***
Sophisticated Cloud Computing	0.0076***	0.0086***	− 0.0317**	− 0.0162***

***, ** and * correspond to *p* < 0.01, *p* < 0.05 and *p* < 0.1 respectively

Furthermore, to examine the association between digital adoption and efficiency expansion across the shipping firms’ efficiency allocation, we employ dummy variables that divide the sample consistent with efficiency quartiles in each division, from lowest to highest initial efficiency levels (Table 8). This table shows the results of the equation where firm-level MFP growth is regressed on growth of the top 5% frontier shipping firms in each division-year cell, the shipping firm’s gap to this threshold, age and size, a dummy for each productivity quartile, and the interaction between digital technology adoption rates and a dummy for each productivity quartile. Quartile 1 indicates the bottom of the allocation, where quartile 4 the upper threshold of it. The last column (1st principal component) exhibits findings of the baseline equation supplemented by an interface term between digitalization (technological tools) and the gap to the frontier.

**Table 8:** The heterogeneous impact of digital adoption on efficiency quartiles

	Broadband Internet Connection	Enterprise Resource Planning	Customer Relationship Management	Ecommerce Website	Sophisticated Cloud Computing	1st Principal Component	1st Principal Component
Frontier growth	0.1955***	0.1865***	0.1925***	0.1905***	0.2055***	0.2095***	0.2245***
	(0.0493)	(0.0482)	(0.0487)	(0.0487)	(0.0492)	(0.0504)	(0.0499)
Gap to frontier	0.0636***	0.0655***	0.0657***	0.0653***	0.0702***	0.0675***	0.0975***
	(0.0302)	(0.0296)	(0.0301)	(0.0298)	(0.0314)	(0.0313)	(0.0225)
Age	− 0.000***	− 0.000***	− 0.000***	− 0.000***	− 0.000***	− 0.000***	− 0.000***
	(5.29e-05)	(5.43e-05)	(5.41e-05)	(5.13e-05)	(5.02e-05)	(5.47e-05)	(5.49e-05)
Employees	0.0093***	0.0092***	0.0097***	0.0093***	0.0107***	0.0111***	0.0109***
	(0.0123)	(0.0111)	(0.0123)	(0.0123)	(0.0124)	(0.0125)	(0.0133)
Quartile 2 (dummy)	− 0.0741***	− 0.0557***	− 0.0682***	− 0.0417**	− 0.0436**	− 0.0497***	
	(0.0229)	(0.0239)	(0.0253)	(0.0245)	(0.0204)	(0.0224)	
Quartile 3 (dummy)	− 0.0809***	− 0.0605***	− 0.0777***	− 0.0463*	− 0.0468*	− 0.0542**	
	(0.0288)	(0.0304)	(0.0302)	(0.0313)	(0.0312)	(0.0299)	
Quartile 4 (dummy)	− 0.0957***	− 0.0754***	− 0.0946***	− 0.0562	− 0.0564	− 0.0659*	
	(0.0368)	(0.0395)	(0.0409)	(0.0403)	(0.0403)	(0.0392)	
Digital technology (Quartile 1)	0.0741***	− 0.0056	0.0895**	0.1115**	0.0563	0.0045**	
	(0.0431)	(0.0557)	(0.0549)	(0.0695)	(0.0846)	(0.0147)	
Digital technology (Quartile 2)	0.1595***	0.0793**	0.1555***	0.0757*	0.0268	0.0048***	
	(0.0437)	(0.0512)	(0.0435)	(0.0554)	(0.0711)	(0.0148)	
Digital technology (Quartile 3)	0.1685***	0.0828**	0.1715***	0.0804**	0.0373	0.0051***	
	(0.0463)	(0.0495)	(0.0402)	(0.0515)	(0.0663)	(0.0672)	
Digital technology (Quartile 4)	0.1805***	0.0985***	0.1915***	0.0783**	0.0368	0.0059***	
	(0.0529)	(0.0491)	(0.0452)	(0.0527)	(0.0628)	(0.0141)	
Digital technology							0.0034***
							(0.0021)
Digital technology * gap to frontier							− 0.0052**
							(0.0024)
R-squared	0.057	0.057	0.057	0.057	0.057	0.060	0.061

***, ** and * correspond to *p* < 0.01, p < 0.05 and *p* < 0.1 respectively. All regressions include division and country-year fixed effects and are grouped at the country-division level. Shipping firms being at the division-year frontier are not included into the regressions. Regressions are based on shipping firm-level data from the 27 EU countries and 2 divisions (NACE Rev.2, Section H) over the period 2015–2020 for firms with a minimum of 10 employees. To expand exposure, unweighted averages of each digital technology indicator have been used over the same period

The findings of this approach are robust and clearly imply the positive correlation between sector-level diffusion of digitalization and efficiency expansion being clearest for shipping firms near the frontier, consistent with findings in previous research, such as Gal et al. (2019) and Dhyne et al. (2018). It is noteworthy that ecommerce website and sophisticated cloud computing are the two out of five technological tools used in our model where low efficiency shipping firms (within quartile 1) benefit more, that the rest of shipping firms; this comes in line with the fact that these two technologies need less investment capital from the firm’s owners’ side (Manganelli and Nicita 2020).

Additionally, to discover whether and in what extent the size of a shipping firm plays vital role concerning returns from digitalization, we have employed relevant regressions segregating the sample firms by size (Table 9). This table shows the results of the equation where firm-level MFP growth is regressed on growth of the top 5% frontier shipping firms in each division-year cell, the shipping firm’s gap to this threshold, age and size, a dummy for each productivity quartile, and the interaction between digital technology adoption rates and a dummy for each productivity quartile. Moreover, size category 1 includes shipping firms with 10–20 workers, size category 2 shipping firms from 21 to 50 workers, size category 3 shipping firms with 51–250 workers, and size category 4 shipping firms with more than 250 workers.

**Table 9:** Observing the impact of digital adoption on efficiency by size category

	Broadband Internet Connection	Enterprise Resource Planning	Customer Relationship Management	Ecommerce Website	Sophisticated Cloud Computing	1st Principal Component
Frontier growth	0.2304***	0.2194***	0.2262***	0.2231***	0.2318***	0.2444***
	(0.0303)	(0.0293)	(0.0298)	(0.0296)	(0.0301)	(0.0313)
Gap to frontier	0.111***	0.111***	0.112***	0.111***	0.114***	0.115***
	(0.0036)	(0.0032)	(0.0035)	(0.0032)	(0.0036)	(0.0044)
Age	− 0.0002***	− 0.0002***	− 0.0002***	− 0.0003***	− 0.0003***	− 0.0003***
	(5.74e-05)	(5.41e-05)	(5.68–05)	(5.55e-05)	(5.69e-05)	(6.47e-05)
Size category 2 (dummy)	0.0237***	0.0213***	0.0239***	0.0286***	0.0292***	0.0266***
	(0.0024)	(0.0038)	(0.0027)	(0.0026)	(0.0019)	(0.0017)
Size category 3 (dummy)	0.0577***	0.0398***	0.0531***	0.0596***	0.0638***	0.0566***
	(0.0071)	(0.0072)	(0.0078)	(0.0063)	(0.0061)	(0.0057)
Size category 4 (dummy)	0.0836***	0.0597***	0.0815***	0.0924***	0.0916***	0.0796***
	(0.0085)	(0.0083)	(0.0085)	(0.0085)	(0.0081)	(0.0074)
Digital technology (Size category 1)	0.1507***	0.0833*	0.1921***	0.1204**	0.0986	0.0252***
	(0.0263)	(0.0345)	(0.0285)	(0.0383)	(0.0502)	(0.0041)
Digital technology (Size category 2)	0.1573***	0.0989**	0.199***	0.1054**	0.0738	0.0249***
	(0.0267)	(0.0331)	(0.0267)	(0.0364)	(0.0479)	(0.0037)
Digital technology (Size category 3)	0.136***	0.129***	0.1967***	0.0922*	0.0271	0.0236***
	(0.0279)	(0.0291)	(0.0271)	(0.0367)	(0.0492)	(0.0032)
Digital technology (Size category 4)	0.1214***	0.136***	0.1751***	− 0.0429	− 0.0198	0.0206***
	(0.0305)	(0.0295)	(0.0281)	(0.0359)	(0.0485)	(0.0029)
R-squared	0.057	0.057	0.058	0.057	0.059	0.060

***, ** and * correspond to *p* < 0.01, *p* < 0.05 and *p* < 0.1 respectively. All regressions include division and country-year fixed effects and are grouped at the country-division level. Shipping firms being at the division-year frontier are not included into the regressions. Regressions are based on shipping firm-level data from the 27 EU countries and 2 divisions (NACE Rev.2, Section H) over the period 2015–2020 for firms with a minimum of 10 employees. To expand exposure, unweighted averages of each digital technology indicator have been used over the same period

What can be interpreted is that size demonstrates less importance than efficiency concerning returns from digitalization. Moreover, some other interesting results to mention are: (1) ecommerce website demonstrates the soundest positive association with efficiency for the size category 1 shipping firms; as stated in Yang et al. (2015), SMEs ecommerce capability could become a rent-generating resource that is not simply copied or replaced, allowing firms to accomplish a viable route to competitive advantage, (2) ecommerce website shows negative association with efficiency for the size category 4 shipping firms, (3) sophisticated cloud computing reveals negative association with efficiency for the size category 4 shipping firms, (4) enterprise resource planning is proved to be strongly positively related to efficiency developments in the size category 4 shipping firms.

## Conclusion and discussion

Given the vital position of the maritime industry in strengthening the global market, the industry’s rather delayed adaptation to a digitalized ecosystem compared to other worldwide industries seems surprising. For those operating in the maritime industry, an industry in which guidelines, procedures, and protocols provide specific directives, adaptive shift could be considered as being rather sluggish. In this study, an attempt has been made to investigate maritime digital adoption and efficiency maturity, shedding light on how industry stakeholders may derive value from data solutions, for making better operational decisions.

Our findings strengthen the concept of digital technologies adoption being commonly linked to considerably greater firm-level efficiency. These findings stand for a variety of technologies (1) Sophisticated Cloud Computing, (2) Broadband Internet Connection, (3) Enterprise Resource Planning, (4) Customer Relationship Management, and (5) Ecommerce Website. This relationship seems clearer in the water transport division, indicating that digital adoption can restructure production practices and, in a way, play a role of an alternative for routine labor input. The relationship amongst the adoption of digital technologies and efficiency seems also clearer for shipping firms that are already highly productive, likely to take advantage of supplementary administrative and technical skills. This proof is consistent with the possibility of digitalization intensifying diffusion in firm-level efficiency results.

The approach in this paper has a number of limitations. First, the data used have been driven from the two available under study Eurostat divisions, under NACE Rev.2 taxonomy, namely water transport activities and warehousing/support activities for transportation. Such analysis does not cover the entire maritime industry. Further research could contain additional non-EU NACE taxonomy codes, broadening the spectrum of the industry’s sectors/segments. Second, since shipping is experiencing increasing pressure to decarbonize its operations and to reduce emissions to air,^8^ further research is needed to improve the understanding of the links between digitalization and decarbonization in the maritime sector, something that we have not gone through in our study. We feel that these two terms should not be treated as though they were two different goals, but we feel that digitalization is a potential enabler towards decarbonization. Third, in connection to the above, one should try to employ the regulatory environment into this model by implementing a sort of regulatory impact indicator, emphasizing on proper data collection, because data of today is really what forms the regulations of tomorrow; this also means that regulations which will come into force over the next years are based on data that have been collected over the preceding years. For instance, the emissions trading scheme is likely to apply to the maritime sector from 2023 onwards; all that was preceded by the EU-MRV (monitoring, reporting, and verification) system^9^ regulations which was all about collecting emissions data from ships. That is partly geared towards the EU being able to set an appropriate quantity of emissions for shipping (ICC 2015).

Though shipping firm efficiency is influenced by a variety of factors, the influence of digital technologies is regularly made in areas implicitly associated to the accounting measures of firm efficiency. One should develop a digital technologies-effect model on the intangible components of efficiency, like product or service quality, customer value-added, and customer loyalty and to investigate which ones are better supported by digital technologies. Additionally, macro-level external resources, such as government incentive policy, could be employed in future research, first to investigate which macro-characteristics are most significant, and second what is the degree on interaction amongst them in determining shipping firms’ capacity to pertain digital technologies for administrative enhancement.

## Data Availability

Authors do not wish to share data due to confidentiality.

## Abbreviations

BT: Blockchain technology
CIT: Computer information technology
ICT: Information and communication technologies
ISM: Interpretive structural modelling
IT: Information technologies
MFP: Multi-factor productivity
NACE: Nomenclature statistique des activités économiques dans la Communauté européenne
OLS: Ordinary least squares
RII: Routine intensity indicator
TAM: Technology acceptance model

## Footnotes

1 Among OECD countries and beyond, the strategy reaction to the digital revolution has been blended. Several are emerging a strategic and pro-active tactic to leveraging its benefits, employing the full range of government policies, whereas others have made fragmentary choices to comprise the effects of particular cases or the effects of new technologies, applications or business models (OECD, 2019).

2 “NACE is the statistical classification of economic activities in the European Community and is the subject of legislation at the European Union level, which imposes the use of the classification uniformly within all the Member States. It is a basic element of the international integrated system of economic classifications, which is based on classifications of the UN Statistical Commission, Eurostat as well as national classifications; all of them strongly related each to the others, allowing the comparability of economic statistics produced worldwide by different institutions. NACE Rev. 2 reflects the technological developments and structural changes of the economy, enabling the modernisation of the Community statistics and contributing, through more comparable and relevant data, to better economic governance at both Community and national level” (Eurostat 2008).

3 ‘Reallocation indicator’ denotes the covariance between efficiency and firm size, firm entry and exit, or ICT (Information and Communication Technologies) related to innovation spillovers (Bartelsman et al. 2017).

4 This indicator is measured as an index and in annual growth rates.

5 The term “advanced firms” refers to shipping firms using advanced technology, namely new technology that performs a new function or improves some function significantly better than other commonly used technology (Galindo-Rueda et al. 2020).

6 According to the Maritime and Port Authority of Singapore (2017), evolution of e-commerce websites and e-payments (as well as the digitalization of logistics) might allow additional SMEs to involve in international trade, enhancing trade development in the longer run. For example, the growth of e-commerce websites in logistics has formulated novel prospects such as “carrier-to-marketplace partnerships and ocean freight platforms”, allowing transporters and shipping lines to empower their asset employment and enhance supply chains.

7 Section H includes the provision of passenger or freight transport, whether scheduled or not, by rail, pipeline, road, water or air and associated activities such as terminal and parking facilities, cargo handling, storage etc. Included in this section is the renting of transport equipment with driver or operator. Also included are postal and courier activities.

8 In April 2018, the IMO adopted an ambitious GHG reduction strategy with a vision to decarbonize shipping as soon as possible within this century. With 2008 as a baseline year, this strategy aims to reduce with at least 50% total GHG emissions from shipping by 2050, while at the same time reducing the average carbon intensity (CO2 per tonne-mile) by at least 40% by 2030, and 70% before mid-century (OECD, 2018b).

9 The European MRV Regulation entered into force in 2015, the first reporting period starts 1 January 2018. Companies operating ships of over 5000GT which carry passengers or cargo for commercial purposes to or from European ports, regardless of the flag they fly, must submit their monitoring plans to an accredited verifier by 31 August 2017.
